# Supplementary material for: Modeling glioblastoma heterogeneity as a dynamic network of cell states
Source: Mol Syst Biol. 2021 Sep 16;17(9):e10105. doi: 10.15252/msb.202010105 (PMC8444284; doi:10.15252/msb.202010105)
Supplement: Supplementary file 6 — Source Data for Figure 5 [file MSB-17-e10105-s004.zip › Figure5A_sourcedata/GSEA_3017/nosigdb_stateF.GseaPreranked.1622795015644/RICHARDS_NOWAKOWSKI_SCIENCE_2017_ORG_UPREG.html]

Details for gene set RICHARDS\_NOWAKOWSKI\_SCIENCE\_2017\_ORG\_UPREG[GSEA]

|  || Dataset | state33017 |
| Phenotype | NoPhenotypeAvailable |
| Upregulated in class | na\_pos |
| GeneSet | RICHARDS\_NOWAKOWSKI\_SCIENCE\_2017\_ORG\_UPREG |
| Enrichment Score (ES) | 0.40606835 |
| Normalized Enrichment Score (NES) | 3.282072 |
| Nominal p-value | 0.0 |
| FDR q-value | 0.0 |
| FWER p-Value | 0.0 |
Table: GSEA Results Summary

  

Fig 1: Enrichment plot: RICHARDS\_NOWAKOWSKI\_SCIENCE\_2017\_ORG\_UPREG      
 Profile of the Running ES Score & Positions of GeneSet Members on the Rank Ordered List

  

| PROBE | GENE SYMBOL | GENE\_TITLE | RANK IN GENE LIST | RANK METRIC SCORE | RUNNING ES | CORE ENRICHMENT || 1 | NEAT1 |  |  | 0 | 2.864 | 0.0125 | Yes |
| 2 | MT3 |  |  | 1 | 2.229 | 0.0222 | Yes |
| 3 | CLU |  |  | 5 | 1.852 | 0.0286 | Yes |
| 4 | SPARCL1 |  |  | 6 | 1.804 | 0.0365 | Yes |
| 5 | ATP1B2 |  |  | 9 | 1.759 | 0.0431 | Yes |
| 6 | ZMAT3 |  |  | 10 | 1.568 | 0.0499 | Yes |
| 7 | HEPACAM |  |  | 13 | 1.356 | 0.0547 | Yes |
| 8 | HOPX |  |  | 14 | 1.345 | 0.0606 | Yes |
| 9 | CST3 |  |  | 15 | 1.273 | 0.0661 | Yes |
| 10 | LGALS3 |  |  | 16 | 1.261 | 0.0716 | Yes |
| 11 | S100B |  |  | 20 | 1.167 | 0.0750 | Yes |
| 12 | SLC1A3 |  |  | 26 | 1.058 | 0.0768 | Yes |
| 13 | SDCBP |  |  | 27 | 1.054 | 0.0814 | Yes |
| 14 | CSPG5 |  |  | 30 | 1.044 | 0.0849 | Yes |
| 15 | LRRC17 |  |  | 31 | 1.044 | 0.0894 | Yes |
| 16 | RPS27L |  |  | 33 | 1.025 | 0.0933 | Yes |
| 17 | ITPR2 |  |  | 34 | 1.024 | 0.0978 | Yes |
| 18 | PLTP |  |  | 35 | 1.020 | 0.1022 | Yes |
| 19 | DKK3 |  |  | 37 | 0.994 | 0.1060 | Yes |
| 20 | IFITM3 |  |  | 38 | 0.987 | 0.1103 | Yes |
| 21 | SLC4A4 |  |  | 40 | 0.980 | 0.1140 | Yes |
| 22 | F3 |  |  | 41 | 0.978 | 0.1183 | Yes |
| 23 | ALDH3A2 |  |  | 43 | 0.958 | 0.1219 | Yes |
| 24 | EDNRB |  |  | 44 | 0.957 | 0.1261 | Yes |
| 25 | SAT1 |  |  | 45 | 0.957 | 0.1303 | Yes |
| 26 | GFAP |  |  | 47 | 0.953 | 0.1339 | Yes |
| 27 | SQSTM1 |  |  | 54 | 0.914 | 0.1345 | Yes |
| 28 | SESN3 |  |  | 56 | 0.908 | 0.1379 | Yes |
| 29 | MLC1 |  |  | 58 | 0.896 | 0.1412 | Yes |
| 30 | ASCC3 |  |  | 60 | 0.873 | 0.1445 | Yes |
| 31 | MYO6 |  |  | 62 | 0.859 | 0.1477 | Yes |
| 32 | SLC1A2 |  |  | 63 | 0.857 | 0.1514 | Yes |
| 33 | SRGAP3 |  |  | 66 | 0.850 | 0.1540 | Yes |
| 34 | GLUL |  |  | 69 | 0.837 | 0.1565 | Yes |
| 35 | GPR155 |  |  | 70 | 0.832 | 0.1601 | Yes |
| 36 | COL4A5 |  |  | 71 | 0.829 | 0.1638 | Yes |
| 37 | CHPT1 |  |  | 73 | 0.818 | 0.1668 | Yes |
| 38 | NDFIP1 |  |  | 74 | 0.814 | 0.1703 | Yes |
| 39 | IFI27L2 |  |  | 76 | 0.812 | 0.1733 | Yes |
| 40 | ITGB5 |  |  | 80 | 0.803 | 0.1751 | Yes |
| 41 | SERPINB6 |  |  | 83 | 0.791 | 0.1774 | Yes |
| 42 | AQP4 |  |  | 84 | 0.789 | 0.1809 | Yes |
| 43 | UBL3 |  |  | 88 | 0.778 | 0.1826 | Yes |
| 44 | LRP1 |  |  | 92 | 0.765 | 0.1842 | Yes |
| 45 | PON2 |  |  | 93 | 0.764 | 0.1875 | Yes |
| 46 | RNF180 |  |  | 101 | 0.751 | 0.1868 | Yes |
| 47 | HES5 |  |  | 102 | 0.749 | 0.1901 | Yes |
| 48 | NDRG4 |  |  | 104 | 0.747 | 0.1928 | Yes |
| 49 | LTBP1 |  |  | 108 | 0.741 | 0.1943 | Yes |
| 50 | PHPT1 |  |  | 109 | 0.738 | 0.1976 | Yes |
| 51 | B2M |  |  | 110 | 0.736 | 0.2008 | Yes |
| 52 | CHN1 |  |  | 112 | 0.730 | 0.2034 | Yes |
| 53 | NPC2 |  |  | 114 | 0.728 | 0.2060 | Yes |
| 54 | NPNT |  |  | 117 | 0.721 | 0.2080 | Yes |
| 55 | NCAN |  |  | 138 | 0.686 | 0.1997 | Yes |
| 56 | TP53I3 |  |  | 139 | 0.686 | 0.2027 | Yes |
| 57 | GNAO1 |  |  | 141 | 0.685 | 0.2051 | Yes |
| 58 | DDB2 |  |  | 143 | 0.682 | 0.2075 | Yes |
| 59 | CDH4 |  |  | 144 | 0.682 | 0.2105 | Yes |
| 60 | TSPAN7 |  |  | 148 | 0.671 | 0.2117 | Yes |
| 61 | PTGFRN |  |  | 151 | 0.664 | 0.2135 | Yes |
| 62 | PIK3C2A |  |  | 156 | 0.653 | 0.2141 | Yes |
| 63 | FAM84A |  |  | 157 | 0.650 | 0.2169 | Yes |
| 64 | DDR1 |  |  | 159 | 0.649 | 0.2192 | Yes |
| 65 | ITM2C |  |  | 160 | 0.649 | 0.2220 | Yes |
| 66 | TOB1 |  |  | 161 | 0.646 | 0.2248 | Yes |
| 67 | PEG3 |  |  | 162 | 0.645 | 0.2277 | Yes |
| 68 | NAMPT |  |  | 168 | 0.634 | 0.2276 | Yes |
| 69 | APC |  |  | 169 | 0.632 | 0.2304 | Yes |
| 70 | CSGALNACT1 |  |  | 172 | 0.628 | 0.2320 | Yes |
| 71 | DTNA |  |  | 176 | 0.617 | 0.2330 | Yes |
| 72 | ST3GAL5 |  |  | 180 | 0.612 | 0.2339 | Yes |
| 73 | ITM2B |  |  | 181 | 0.610 | 0.2366 | Yes |
| 74 | PLOD2 |  |  | 183 | 0.607 | 0.2387 | Yes |
| 75 | UBE2H |  |  | 189 | 0.601 | 0.2385 | Yes |
| 76 | CD44 |  |  | 191 | 0.600 | 0.2405 | Yes |
| 77 | FUT8 |  |  | 194 | 0.593 | 0.2420 | Yes |
| 78 | TIMP2 |  |  | 196 | 0.590 | 0.2440 | Yes |
| 79 | HERPUD1 |  |  | 197 | 0.590 | 0.2466 | Yes |
| 80 | C1orf61 |  |  | 202 | 0.587 | 0.2469 | Yes |
| 81 | ENHO |  |  | 203 | 0.586 | 0.2494 | Yes |
| 82 | HIGD1A |  |  | 212 | 0.580 | 0.2474 | Yes |
| 83 | HSPA4L |  |  | 215 | 0.579 | 0.2488 | Yes |
| 84 | RRAGD |  |  | 218 | 0.578 | 0.2502 | Yes |
| 85 | PDGFC |  |  | 219 | 0.577 | 0.2527 | Yes |
| 86 | PRUNE2 |  |  | 221 | 0.575 | 0.2547 | Yes |
| 87 | PDLIM5 |  |  | 223 | 0.572 | 0.2566 | Yes |
| 88 | SLC2A3 |  |  | 228 | 0.569 | 0.2568 | Yes |
| 89 | HTRA1 |  |  | 229 | 0.568 | 0.2593 | Yes |
| 90 | SCHIP1 |  |  | 233 | 0.566 | 0.2601 | Yes |
| 91 | LIFR |  |  | 239 | 0.562 | 0.2597 | Yes |
| 92 | MEGF10 |  |  | 240 | 0.560 | 0.2621 | Yes |
| 93 | CA2 |  |  | 243 | 0.558 | 0.2634 | Yes |
| 94 | PHYHIPL |  |  | 245 | 0.557 | 0.2653 | Yes |
| 95 | PBXIP1 |  |  | 250 | 0.554 | 0.2654 | Yes |
| 96 | PEPD |  |  | 253 | 0.550 | 0.2667 | Yes |
| 97 | MAGED1 |  |  | 258 | 0.545 | 0.2668 | Yes |
| 98 | S100A13 |  |  | 261 | 0.544 | 0.2681 | Yes |
| 99 | ARL6IP5 |  |  | 264 | 0.542 | 0.2693 | Yes |
| 100 | GPR137B |  |  | 265 | 0.541 | 0.2717 | Yes |
| 101 | FAM3C |  |  | 266 | 0.541 | 0.2740 | Yes |
| 102 | CRISPLD1 |  |  | 268 | 0.538 | 0.2758 | Yes |
| 103 | TFRC |  |  | 270 | 0.537 | 0.2776 | Yes |
| 104 | HMGCS1 |  |  | 271 | 0.537 | 0.2799 | Yes |
| 105 | SPAG16 |  |  | 273 | 0.535 | 0.2817 | Yes |
| 106 | FAM19A5 |  |  | 277 | 0.527 | 0.2823 | Yes |
| 107 | SLC25A18 |  |  | 280 | 0.525 | 0.2834 | Yes |
| 108 | FAM162A |  |  | 282 | 0.524 | 0.2852 | Yes |
| 109 | TPP1 |  |  | 286 | 0.521 | 0.2857 | Yes |
| 110 | CCNG1 |  |  | 292 | 0.515 | 0.2852 | Yes |
| 111 | BCAN |  |  | 302 | 0.510 | 0.2823 | Yes |
| 112 | ATP6V0E1 |  |  | 303 | 0.510 | 0.2845 | Yes |
| 113 | TRIM5 |  |  | 305 | 0.508 | 0.2862 | Yes |
| 114 | LAMP2 |  |  | 308 | 0.506 | 0.2872 | Yes |
| 115 | PTPRZ1 |  |  | 310 | 0.505 | 0.2889 | Yes |
| 116 | GSTK1 |  |  | 312 | 0.503 | 0.2905 | Yes |
| 117 | TMEM47 |  |  | 316 | 0.502 | 0.2910 | Yes |
| 118 | ACYP2 |  |  | 317 | 0.502 | 0.2932 | Yes |
| 119 | NUDT4 |  |  | 318 | 0.502 | 0.2954 | Yes |
| 120 | HES1 |  |  | 320 | 0.501 | 0.2970 | Yes |
| 121 | IL6ST |  |  | 321 | 0.500 | 0.2992 | Yes |
| 122 | CHMP4B |  |  | 322 | 0.500 | 0.3014 | Yes |
| 123 | HS6ST1 |  |  | 323 | 0.499 | 0.3035 | Yes |
| 124 | CNTNAP3B |  |  | 324 | 0.498 | 0.3057 | Yes |
| 125 | PSAT1 |  |  | 329 | 0.496 | 0.3056 | Yes |
| 126 | RNF13 |  |  | 330 | 0.496 | 0.3078 | Yes |
| 127 | GPC6 |  |  | 332 | 0.495 | 0.3094 | Yes |
| 128 | SEMA5A |  |  | 336 | 0.494 | 0.3098 | Yes |
| 129 | PLD3 |  |  | 337 | 0.494 | 0.3120 | Yes |
| 130 | OLFM2 |  |  | 343 | 0.490 | 0.3113 | Yes |
| 131 | ITGAV |  |  | 344 | 0.490 | 0.3134 | Yes |
| 132 | ACSBG1 |  |  | 345 | 0.490 | 0.3156 | Yes |
| 133 | GSTM3 |  |  | 348 | 0.488 | 0.3166 | Yes |
| 134 | SYT11 |  |  | 352 | 0.486 | 0.3170 | Yes |
| 135 | SCP2 |  |  | 353 | 0.485 | 0.3191 | Yes |
| 136 | ATP2B4 |  |  | 356 | 0.485 | 0.3201 | Yes |
| 137 | CYSTM1 |  |  | 358 | 0.484 | 0.3216 | Yes |
| 138 | PSPH |  |  | 359 | 0.484 | 0.3238 | Yes |
| 139 | PDCD4 |  |  | 360 | 0.484 | 0.3259 | Yes |
| 140 | PAM |  |  | 361 | 0.483 | 0.3280 | Yes |
| 141 | ABAT |  |  | 362 | 0.483 | 0.3301 | Yes |
| 142 | LIPA |  |  | 364 | 0.483 | 0.3316 | Yes |
| 143 | ID2 |  |  | 366 | 0.483 | 0.3332 | Yes |
| 144 | HSPA5 |  |  | 373 | 0.480 | 0.3319 | Yes |
| 145 | GATM |  |  | 375 | 0.480 | 0.3334 | Yes |
| 146 | CTSL |  |  | 376 | 0.479 | 0.3355 | Yes |
| 147 | ALDOA |  |  | 377 | 0.479 | 0.3376 | Yes |
| 148 | ARHGAP18 |  |  | 378 | 0.479 | 0.3397 | Yes |
| 149 | SEMA6A |  |  | 381 | 0.478 | 0.3406 | Yes |
| 150 | LRRTM3 |  |  | 387 | 0.476 | 0.3399 | Yes |
| 151 | COMT |  |  | 396 | 0.471 | 0.3374 | Yes |
| 152 | CES2 |  |  | 400 | 0.470 | 0.3377 | Yes |
| 153 | METTL7A |  |  | 403 | 0.469 | 0.3386 | Yes |
| 154 | MAGED2 |  |  | 405 | 0.467 | 0.3401 | Yes |
| 155 | ITFG1 |  |  | 406 | 0.467 | 0.3422 | Yes |
| 156 | ZHX1 |  |  | 409 | 0.466 | 0.3431 | Yes |
| 157 | VCAN |  |  | 410 | 0.466 | 0.3451 | Yes |
| 158 | SLC3A2 |  |  | 411 | 0.466 | 0.3471 | Yes |
| 159 | PSAP |  |  | 418 | 0.463 | 0.3458 | Yes |
| 160 | PRDX5 |  |  | 419 | 0.463 | 0.3478 | Yes |
| 161 | DNASE2 |  |  | 420 | 0.462 | 0.3498 | Yes |
| 162 | FAM171B |  |  | 422 | 0.462 | 0.3512 | Yes |
| 163 | PRCP |  |  | 426 | 0.460 | 0.3516 | Yes |
| 164 | EMC10 |  |  | 427 | 0.460 | 0.3536 | Yes |
| 165 | TKT |  |  | 431 | 0.459 | 0.3539 | Yes |
| 166 | NADK2 |  |  | 437 | 0.456 | 0.3530 | Yes |
| 167 | RPS6KA5 |  |  | 438 | 0.456 | 0.3550 | Yes |
| 168 | LAMP1 |  |  | 440 | 0.455 | 0.3564 | Yes |
| 169 | TRPS1 |  |  | 441 | 0.455 | 0.3584 | Yes |
| 170 | MAP1LC3B |  |  | 445 | 0.453 | 0.3587 | Yes |
| 171 | MAST4 |  |  | 446 | 0.451 | 0.3607 | Yes |
| 172 | DDIT3 |  |  | 447 | 0.451 | 0.3626 | Yes |
| 173 | HSD17B12 |  |  | 453 | 0.449 | 0.3618 | Yes |
| 174 | TTYH1 |  |  | 457 | 0.448 | 0.3620 | Yes |
| 175 | CROT |  |  | 460 | 0.447 | 0.3628 | Yes |
| 176 | FHL1 |  |  | 467 | 0.445 | 0.3614 | Yes |
| 177 | TIPARP |  |  | 472 | 0.444 | 0.3610 | Yes |
| 178 | JUND |  |  | 478 | 0.442 | 0.3601 | Yes |
| 179 | RFTN2 |  |  | 485 | 0.439 | 0.3586 | Yes |
| 180 | SCOC |  |  | 487 | 0.439 | 0.3600 | Yes |
| 181 | LPIN2 |  |  | 488 | 0.438 | 0.3619 | Yes |
| 182 | HEATR5A |  |  | 491 | 0.438 | 0.3627 | Yes |
| 183 | LRP10 |  |  | 492 | 0.438 | 0.3646 | Yes |
| 184 | HEY1 |  |  | 496 | 0.436 | 0.3648 | Yes |
| 185 | NDUFB5 |  |  | 498 | 0.435 | 0.3661 | Yes |
| 186 | FAT3 |  |  | 501 | 0.434 | 0.3669 | Yes |
| 187 | OBSL1 |  |  | 502 | 0.434 | 0.3688 | Yes |
| 188 | FZD7 |  |  | 504 | 0.434 | 0.3701 | Yes |
| 189 | AHCYL2 |  |  | 508 | 0.432 | 0.3703 | Yes |
| 190 | YBX3 |  |  | 509 | 0.431 | 0.3722 | Yes |
| 191 | ZNF561 |  |  | 511 | 0.430 | 0.3735 | Yes |
| 192 | ATP6AP2 |  |  | 514 | 0.430 | 0.3742 | Yes |
| 193 | FGFR3 |  |  | 515 | 0.429 | 0.3761 | Yes |
| 194 | PEA15 |  |  | 521 | 0.427 | 0.3752 | Yes |
| 195 | CA12 |  |  | 522 | 0.426 | 0.3770 | Yes |
| 196 | GNPDA1 |  |  | 523 | 0.425 | 0.3789 | Yes |
| 197 | LAMB2 |  |  | 524 | 0.425 | 0.3807 | Yes |
| 198 | ELOVL2 |  |  | 527 | 0.424 | 0.3814 | Yes |
| 199 | LGALS3BP |  |  | 528 | 0.424 | 0.3833 | Yes |
| 200 | PELI2 |  |  | 529 | 0.422 | 0.3851 | Yes |
| 201 | TFPI |  |  | 531 | 0.421 | 0.3864 | Yes |
| 202 | FKBP9 |  |  | 533 | 0.419 | 0.3877 | Yes |
| 203 | TMEM9B |  |  | 539 | 0.417 | 0.3867 | Yes |
| 204 | ADAM10 |  |  | 540 | 0.417 | 0.3885 | Yes |
| 205 | PARP14 |  |  | 541 | 0.417 | 0.3903 | Yes |
| 206 | TANC2 |  |  | 542 | 0.417 | 0.3921 | Yes |
| 207 | GOLM1 |  |  | 545 | 0.417 | 0.3928 | Yes |
| 208 | MOXD1 |  |  | 549 | 0.415 | 0.3929 | Yes |
| 209 | PNPLA8 |  |  | 551 | 0.414 | 0.3942 | Yes |
| 210 | GPM6B |  |  | 553 | 0.413 | 0.3954 | Yes |
| 211 | PMP2 |  |  | 562 | 0.411 | 0.3927 | Yes |
| 212 | BICD1 |  |  | 563 | 0.410 | 0.3944 | Yes |
| 213 | IGFBP5 |  |  | 564 | 0.410 | 0.3962 | Yes |
| 214 | GRB14 |  |  | 566 | 0.409 | 0.3974 | Yes |
| 215 | RAB6A |  |  | 571 | 0.407 | 0.3970 | Yes |
| 216 | CNN3 |  |  | 576 | 0.407 | 0.3965 | Yes |
| 217 | SORT1 |  |  | 577 | 0.406 | 0.3982 | Yes |
| 218 | RAB31 |  |  | 580 | 0.404 | 0.3989 | Yes |
| 219 | NDN |  |  | 585 | 0.403 | 0.3984 | Yes |
| 220 | FOS |  |  | 592 | 0.401 | 0.3967 | Yes |
| 221 | VGLL4 |  |  | 594 | 0.400 | 0.3979 | Yes |
| 222 | SDC2 |  |  | 596 | 0.399 | 0.3991 | Yes |
| 223 | ARAP2 |  |  | 599 | 0.399 | 0.3997 | Yes |
| 224 | NBR1 |  |  | 601 | 0.397 | 0.4008 | Yes |
| 225 | CRIM1 |  |  | 602 | 0.397 | 0.4026 | Yes |
| 226 | MAP1A |  |  | 603 | 0.396 | 0.4043 | Yes |
| 227 | LSAMP |  |  | 611 | 0.394 | 0.4021 | Yes |
| 228 | TNC |  |  | 613 | 0.393 | 0.4032 | Yes |
| 229 | TMTC2 |  |  | 619 | 0.392 | 0.4021 | Yes |
| 230 | REXO2 |  |  | 620 | 0.392 | 0.4038 | Yes |
| 231 | FKBP2 |  |  | 622 | 0.391 | 0.4049 | Yes |
| 232 | NDUFB3 |  |  | 629 | 0.389 | 0.4032 | Yes |
| 233 | TAOK3 |  |  | 631 | 0.389 | 0.4044 | Yes |
| 234 | RAB5A |  |  | 632 | 0.389 | 0.4061 | Yes |
| 235 | TMEM14A |  |  | 647 | 0.386 | 0.3998 | No |
| 236 | AIG1 |  |  | 651 | 0.385 | 0.3998 | No |
| 237 | ID4 |  |  | 659 | 0.383 | 0.3975 | No |
| 238 | NFE2L2 |  |  | 663 | 0.381 | 0.3975 | No |
| 239 | CDH10 |  |  | 669 | 0.379 | 0.3963 | No |
| 240 | ADD3 |  |  | 674 | 0.378 | 0.3957 | No |
| 241 | ASTN1 |  |  | 679 | 0.375 | 0.3950 | No |
| 242 | RAB6B |  |  | 688 | 0.374 | 0.3921 | No |
| 243 | DNAJC3 |  |  | 689 | 0.374 | 0.3938 | No |
| 244 | LRRCC1 |  |  | 690 | 0.374 | 0.3954 | No |
| 245 | RGS16 |  |  | 691 | 0.373 | 0.3970 | No |
| 246 | ZFYVE21 |  |  | 694 | 0.373 | 0.3975 | No |
| 247 | SH3BGRL |  |  | 695 | 0.373 | 0.3991 | No |
| 248 | KCNK10 |  |  | 697 | 0.372 | 0.4002 | No |
| 249 | FABP7 |  |  | 701 | 0.371 | 0.4001 | No |
| 250 | SGK3 |  |  | 709 | 0.369 | 0.3978 | No |
| 251 | C5orf30 |  |  | 712 | 0.368 | 0.3982 | No |
| 252 | CSTB |  |  | 714 | 0.368 | 0.3993 | No |
| 253 | SIPA1L1 |  |  | 717 | 0.367 | 0.3997 | No |
| 254 | FAM107A |  |  | 718 | 0.367 | 0.4013 | No |
| 255 | ADAM9 |  |  | 722 | 0.365 | 0.4012 | No |
| 256 | SNN |  |  | 732 | 0.363 | 0.3977 | No |
| 257 | GNPTG |  |  | 734 | 0.363 | 0.3987 | No |
| 258 | SC5D |  |  | 737 | 0.362 | 0.3992 | No |
| 259 | ZHX3 |  |  | 741 | 0.361 | 0.3991 | No |
| 260 | COQ10B |  |  | 749 | 0.359 | 0.3967 | No |
| 261 | MASP1 |  |  | 755 | 0.357 | 0.3954 | No |
| 262 | CDIP1 |  |  | 768 | 0.354 | 0.3901 | No |
| 263 | LRP4 |  |  | 773 | 0.353 | 0.3894 | No |
| 264 | FSTL1 |  |  | 774 | 0.352 | 0.3909 | No |
| 265 | MSI2 |  |  | 780 | 0.352 | 0.3896 | No |
| 266 | SLC12A2 |  |  | 782 | 0.351 | 0.3906 | No |
| 267 | RORA |  |  | 783 | 0.351 | 0.3921 | No |
| 268 | SLC15A2 |  |  | 784 | 0.351 | 0.3937 | No |
| 269 | HSD17B7 |  |  | 791 | 0.349 | 0.3918 | No |
| 270 | C3orf70 |  |  | 794 | 0.348 | 0.3922 | No |
| 271 | TMTC4 |  |  | 795 | 0.348 | 0.3937 | No |
| 272 | FBXO32 |  |  | 800 | 0.347 | 0.3929 | No |
| 273 | FAM84B |  |  | 804 | 0.346 | 0.3927 | No |
| 274 | BDH2 |  |  | 806 | 0.346 | 0.3937 | No |
| 275 | NEDD9 |  |  | 817 | 0.344 | 0.3895 | No |
| 276 | STIM2 |  |  | 818 | 0.344 | 0.3910 | No |
| 277 | PPARGC1A |  |  | 821 | 0.343 | 0.3914 | No |
| 278 | OSBPL1A |  |  | 828 | 0.341 | 0.3895 | No |
| 279 | RGL1 |  |  | 831 | 0.341 | 0.3898 | No |
| 280 | DAG1 |  |  | 834 | 0.340 | 0.3902 | No |
| 281 | TSPAN6 |  |  | 838 | 0.340 | 0.3900 | No |
| 282 | VEGFA |  |  | 840 | 0.339 | 0.3909 | No |
| 283 | RHOQ |  |  | 843 | 0.339 | 0.3912 | No |
| 284 | RFX3 |  |  | 844 | 0.338 | 0.3927 | No |
| 285 | NTPCR |  |  | 847 | 0.337 | 0.3930 | No |
| 286 | RCN1 |  |  | 849 | 0.337 | 0.3939 | No |
| 287 | ZFP36L2 |  |  | 850 | 0.336 | 0.3954 | No |
| 288 | LAPTM4A |  |  | 854 | 0.335 | 0.3952 | No |
| 289 | MSMO1 |  |  | 860 | 0.333 | 0.3938 | No |
| 290 | RHOJ |  |  | 874 | 0.331 | 0.3878 | No |
| 291 | ACER3 |  |  | 877 | 0.330 | 0.3882 | No |
| 292 | ZFHX4 |  |  | 878 | 0.330 | 0.3896 | No |
| 293 | STAT3 |  |  | 880 | 0.330 | 0.3905 | No |
| 294 | L3HYPDH |  |  | 883 | 0.329 | 0.3908 | No |
| 295 | ARHGEF26 |  |  | 885 | 0.328 | 0.3916 | No |
| 296 | CRYL1 |  |  | 891 | 0.327 | 0.3902 | No |
| 297 | SCRN1 |  |  | 904 | 0.325 | 0.3848 | No |
| 298 | BACE1 |  |  | 907 | 0.324 | 0.3851 | No |
| 299 | IGDCC4 |  |  | 911 | 0.324 | 0.3848 | No |
| 300 | ACOX1 |  |  | 912 | 0.324 | 0.3863 | No |
| 301 | ADCYAP1R1 |  |  | 913 | 0.324 | 0.3877 | No |
| 302 | PLEC |  |  | 914 | 0.324 | 0.3891 | No |
| 303 | NCAM2 |  |  | 915 | 0.324 | 0.3905 | No |
| 304 | RIT1 |  |  | 916 | 0.324 | 0.3919 | No |
| 305 | PDCD6 |  |  | 917 | 0.323 | 0.3933 | No |
| 306 | GDPD2 |  |  | 921 | 0.323 | 0.3930 | No |
| 307 | IFI6 |  |  | 922 | 0.323 | 0.3944 | No |
| 308 | NKAIN4 |  |  | 923 | 0.322 | 0.3958 | No |
| 309 | BHLHE40 |  |  | 928 | 0.321 | 0.3950 | No |
| 310 | PNMA1 |  |  | 931 | 0.320 | 0.3952 | No |
| 311 | CLCN3 |  |  | 937 | 0.320 | 0.3938 | No |
| 312 | ATP10D |  |  | 938 | 0.320 | 0.3952 | No |
| 313 | TMBIM4 |  |  | 943 | 0.319 | 0.3943 | No |
| 314 | TLN2 |  |  | 944 | 0.318 | 0.3957 | No |
| 315 | UNC50 |  |  | 945 | 0.318 | 0.3971 | No |
| 316 | CD81 |  |  | 947 | 0.318 | 0.3979 | No |
| 317 | USP2 |  |  | 953 | 0.317 | 0.3965 | No |
| 318 | GAB1 |  |  | 958 | 0.316 | 0.3956 | No |
| 319 | SLITRK2 |  |  | 967 | 0.315 | 0.3924 | No |
| 320 | FAT1 |  |  | 968 | 0.315 | 0.3938 | No |
| 321 | ITGB8 |  |  | 970 | 0.314 | 0.3946 | No |
| 322 | MOSPD2 |  |  | 980 | 0.313 | 0.3909 | No |
| 323 | ZMAT1 |  |  | 983 | 0.313 | 0.3911 | No |
| 324 | ALDH6A1 |  |  | 987 | 0.311 | 0.3908 | No |
| 325 | NIPSNAP3A |  |  | 991 | 0.311 | 0.3904 | No |
| 326 | EMC2 |  |  | 1001 | 0.310 | 0.3867 | No |
| 327 | CXCR4 |  |  | 1003 | 0.309 | 0.3875 | No |
| 328 | TMED7 |  |  | 1009 | 0.308 | 0.3860 | No |
| 329 | ARID5B |  |  | 1014 | 0.307 | 0.3850 | No |
| 330 | SHISA5 |  |  | 1016 | 0.307 | 0.3858 | No |
| 331 | SIRT2 |  |  | 1018 | 0.307 | 0.3866 | No |
| 332 | LRPAP1 |  |  | 1022 | 0.306 | 0.3862 | No |
| 333 | DUSP3 |  |  | 1023 | 0.306 | 0.3876 | No |
| 334 | SCRG1 |  |  | 1032 | 0.304 | 0.3843 | No |
| 335 | PCGF5 |  |  | 1045 | 0.301 | 0.3789 | No |
| 336 | CTNND2 |  |  | 1048 | 0.300 | 0.3790 | No |
| 337 | UBE2E1 |  |  | 1050 | 0.300 | 0.3798 | No |
| 338 | ARHGEF6 |  |  | 1054 | 0.299 | 0.3794 | No |
| 339 | NRBP2 |  |  | 1060 | 0.298 | 0.3778 | No |
| 340 | CACHD1 |  |  | 1061 | 0.298 | 0.3791 | No |
| 341 | CUTA |  |  | 1062 | 0.298 | 0.3805 | No |
| 342 | MEGF8 |  |  | 1065 | 0.298 | 0.3806 | No |
| 343 | CD9 |  |  | 1070 | 0.297 | 0.3796 | No |
| 344 | TSPAN3 |  |  | 1071 | 0.297 | 0.3809 | No |
| 345 | PLEKHA5 |  |  | 1077 | 0.296 | 0.3794 | No |
| 346 | DHRS7 |  |  | 1079 | 0.296 | 0.3801 | No |
| 347 | C1orf122 |  |  | 1081 | 0.296 | 0.3809 | No |
| 348 | SPECC1 |  |  | 1089 | 0.294 | 0.3782 | No |
| 349 | RNF213 |  |  | 1091 | 0.294 | 0.3789 | No |
| 350 | THAP5 |  |  | 1092 | 0.294 | 0.3802 | No |
| 351 | SCARA3 |  |  | 1096 | 0.293 | 0.3797 | No |
| 352 | DNER |  |  | 1104 | 0.292 | 0.3770 | No |
| 353 | TK2 |  |  | 1115 | 0.290 | 0.3726 | No |
| 354 | HBP1 |  |  | 1125 | 0.288 | 0.3688 | No |
| 355 | SLC7A11 |  |  | 1131 | 0.288 | 0.3672 | No |
| 356 | SYPL1 |  |  | 1137 | 0.287 | 0.3656 | No |
| 357 | LINC01278 |  |  | 1142 | 0.286 | 0.3646 | No |
| 358 | SEMA5B |  |  | 1147 | 0.285 | 0.3636 | No |
| 359 | SNX3 |  |  | 1150 | 0.285 | 0.3637 | No |
| 360 | EFNB1 |  |  | 1152 | 0.285 | 0.3644 | No |
| 361 | DMD |  |  | 1153 | 0.285 | 0.3656 | No |
| 362 | GPC1 |  |  | 1162 | 0.284 | 0.3623 | No |
| 363 | STXBP3 |  |  | 1164 | 0.283 | 0.3630 | No |
| 364 | MRPS28 |  |  | 1171 | 0.283 | 0.3608 | No |
| 365 | LAMA1 |  |  | 1173 | 0.283 | 0.3615 | No |
| 366 | ZBTB20 |  |  | 1181 | 0.281 | 0.3587 | No |
| 367 | KCNN3 |  |  | 1182 | 0.281 | 0.3600 | No |
| 368 | CTNNB1 |  |  | 1190 | 0.280 | 0.3572 | No |
| 369 | DOK5 |  |  | 1193 | 0.279 | 0.3573 | No |
| 370 | RGS20 |  |  | 1197 | 0.279 | 0.3568 | No |
| 371 | CKB |  |  | 1200 | 0.279 | 0.3569 | No |
| 372 | PCBD1 |  |  | 1202 | 0.279 | 0.3576 | No |
| 373 | JAM2 |  |  | 1204 | 0.279 | 0.3582 | No |
| 374 | FZD3 |  |  | 1214 | 0.277 | 0.3543 | No |
| 375 | KIF1A |  |  | 1220 | 0.277 | 0.3527 | No |
| 376 | CAPZA2 |  |  | 1223 | 0.276 | 0.3528 | No |
| 377 | CTNNA1 |  |  | 1228 | 0.276 | 0.3517 | No |
| 378 | FEZ2 |  |  | 1232 | 0.276 | 0.3512 | No |
| 379 | TCEAL1 |  |  | 1235 | 0.276 | 0.3513 | No |
| 380 | MAPK1 |  |  | 1250 | 0.274 | 0.3445 | No |
| 381 | CHP1 |  |  | 1251 | 0.274 | 0.3457 | No |
| 382 | USP8 |  |  | 1253 | 0.274 | 0.3464 | No |
| 383 | STARD4 |  |  | 1254 | 0.274 | 0.3476 | No |
| 384 | ALDH7A1 |  |  | 1255 | 0.273 | 0.3487 | No |
| 385 | C2orf76 |  |  | 1257 | 0.273 | 0.3494 | No |
| 386 | NBPF14 |  |  | 1258 | 0.273 | 0.3506 | No |
| 387 | VCAM1 |  |  | 1266 | 0.272 | 0.3478 | No |
| 388 | SBDS |  |  | 1272 | 0.272 | 0.3461 | No |
| 389 | SALL2 |  |  | 1274 | 0.271 | 0.3467 | No |
| 390 | KIF1B |  |  | 1276 | 0.271 | 0.3474 | No |
| 391 | NCKAP5 |  |  | 1279 | 0.271 | 0.3474 | No |
| 392 | LMBRD1 |  |  | 1280 | 0.271 | 0.3486 | No |
| 393 | NLGN3 |  |  | 1292 | 0.269 | 0.3435 | No |
| 394 | SLC25A23 |  |  | 1307 | 0.267 | 0.3368 | No |
| 395 | IRF2BP2 |  |  | 1311 | 0.267 | 0.3362 | No |
| 396 | DPYSL5 |  |  | 1312 | 0.267 | 0.3374 | No |
| 397 | ABHD4 |  |  | 1313 | 0.267 | 0.3386 | No |
| 398 | SDF2 |  |  | 1318 | 0.266 | 0.3374 | No |
| 399 | RFX4 |  |  | 1320 | 0.266 | 0.3380 | No |
| 400 | GLUD1 |  |  | 1334 | 0.264 | 0.3318 | No |
| 401 | MAPK8IP1 |  |  | 1340 | 0.263 | 0.3301 | No |
| 402 | ZDHHC2 |  |  | 1342 | 0.262 | 0.3307 | No |
| 403 | PARP9 |  |  | 1344 | 0.262 | 0.3313 | No |
| 404 | WASF3 |  |  | 1345 | 0.262 | 0.3324 | No |
| 405 | ST8SIA1 |  |  | 1346 | 0.262 | 0.3336 | No |
| 406 | GAPDH |  |  | 1361 | 0.261 | 0.3268 | No |
| 407 | SNX7 |  |  | 1363 | 0.261 | 0.3273 | No |
| 408 | SECISBP2L |  |  | 1366 | 0.261 | 0.3273 | No |
| 409 | SLCO1C1 |  |  | 1372 | 0.260 | 0.3256 | No |
| 410 | CDC14B |  |  | 1378 | 0.259 | 0.3239 | No |
| 411 | ABCD3 |  |  | 1381 | 0.259 | 0.3239 | No |
| 412 | ABHD3 |  |  | 1382 | 0.259 | 0.3251 | No |
| 413 | GEM |  |  | 1391 | 0.257 | 0.3217 | No |
| 414 | ITGA2 |  |  | 1395 | 0.257 | 0.3211 | No |
| 415 | EEPD1 |  |  | 1397 | 0.257 | 0.3216 | No |
| 416 | WSCD1 |  |  | 1401 | 0.256 | 0.3210 | No |
| 417 | WDFY3 |  |  | 1402 | 0.256 | 0.3222 | No |
| 418 | PDE4B |  |  | 1404 | 0.256 | 0.3227 | No |
| 419 | MEIS1 |  |  | 1407 | 0.256 | 0.3227 | No |
| 420 | PDHB |  |  | 1413 | 0.255 | 0.3210 | No |
| 421 | NOTCH2 |  |  | 1420 | 0.255 | 0.3187 | No |
| 422 | GLDC |  |  | 1425 | 0.255 | 0.3175 | No |
| 423 | CALCOCO2 |  |  | 1447 | 0.253 | 0.3067 | No |
| 424 | AGGF1 |  |  | 1454 | 0.252 | 0.3044 | No |
| 425 | TTC9 |  |  | 1462 | 0.251 | 0.3015 | No |
| 426 | PEX2 |  |  | 1463 | 0.251 | 0.3026 | No |
| 427 | JUNB |  |  | 1466 | 0.251 | 0.3026 | No |
| 428 | BBS2 |  |  | 1472 | 0.250 | 0.3008 | No |
| 429 | RPA2 |  |  | 1490 | -0.256 | 0.2923 | No |
| 430 | HSPH1 |  |  | 1495 | -0.258 | 0.2912 | No |
| 431 | ITGB1BP1 |  |  | 1501 | -0.259 | 0.2895 | No |
| 432 | ANXA2 |  |  | 1511 | -0.263 | 0.2855 | No |
| 433 | ACAT2 |  |  | 1512 | -0.263 | 0.2867 | No |
| 434 | HSP90AA1 |  |  | 1535 | -0.268 | 0.2754 | No |
| 435 | PRRC2A |  |  | 1555 | -0.273 | 0.2658 | No |
| 436 | PSMB6 |  |  | 1558 | -0.274 | 0.2658 | No |
| 437 | CREB5 |  |  | 1561 | -0.275 | 0.2659 | No |
| 438 | NAP1L4 |  |  | 1568 | -0.277 | 0.2637 | No |
| 439 | PFN1 |  |  | 1582 | -0.282 | 0.2576 | No |
| 440 | HBEGF |  |  | 1583 | -0.282 | 0.2588 | No |
| 441 | ZFP36L1 |  |  | 1590 | -0.284 | 0.2566 | No |
| 442 | PRKDC |  |  | 1594 | -0.285 | 0.2562 | No |
| 443 | LHX2 |  |  | 1597 | -0.285 | 0.2563 | No |
| 444 | NELFE |  |  | 1631 | -0.294 | 0.2389 | No |
| 445 | CEP128 |  |  | 1657 | -0.304 | 0.2260 | No |
| 446 | ANAPC11 |  |  | 1667 | -0.306 | 0.2222 | No |
| 447 | ENO1 |  |  | 1673 | -0.308 | 0.2207 | No |
| 448 | TSR1 |  |  | 1677 | -0.309 | 0.2204 | No |
| 449 | RDX |  |  | 1678 | -0.310 | 0.2217 | No |
| 450 | FADS1 |  |  | 1682 | -0.312 | 0.2214 | No |
| 451 | TACC1 |  |  | 1701 | -0.319 | 0.2126 | No |
| 452 | NEDD1 |  |  | 1710 | -0.322 | 0.2095 | No |
| 453 | PARP1 |  |  | 1713 | -0.323 | 0.2097 | No |
| 454 | PHLDA1 |  |  | 1718 | -0.324 | 0.2089 | No |
| 455 | PLEKHJ1 |  |  | 1720 | -0.326 | 0.2097 | No |
| 456 | UGP2 |  |  | 1725 | -0.327 | 0.2089 | No |
| 457 | BRD7 |  |  | 1730 | -0.330 | 0.2081 | No |
| 458 | DSEL |  |  | 1735 | -0.333 | 0.2072 | No |
| 459 | DDX1 |  |  | 1741 | -0.336 | 0.2059 | No |
| 460 | HSPD1 |  |  | 1742 | -0.336 | 0.2073 | No |
| 461 | ZYX |  |  | 1745 | -0.338 | 0.2077 | No |
| 462 | GLO1 |  |  | 1755 | -0.342 | 0.2041 | No |
| 463 | TMEM107 |  |  | 1789 | -0.357 | 0.1869 | No |
| 464 | LGALS1 |  |  | 1799 | -0.362 | 0.1834 | No |
| 465 | NES |  |  | 1811 | -0.370 | 0.1788 | No |
| 466 | RHNO1 |  |  | 1824 | -0.377 | 0.1736 | No |
| 467 | XRCC6 |  |  | 1825 | -0.377 | 0.1753 | No |
| 468 | GJC1 |  |  | 1829 | -0.379 | 0.1752 | No |
| 469 | LDHA |  |  | 1833 | -0.380 | 0.1752 | No |
| 470 | BARD1 |  |  | 1839 | -0.384 | 0.1740 | No |
| 471 | CREM |  |  | 1842 | -0.385 | 0.1746 | No |
| 472 | SQLE |  |  | 1863 | -0.396 | 0.1649 | No |
| 473 | PPP1CA |  |  | 1872 | -0.399 | 0.1621 | No |
| 474 | NAE1 |  |  | 1881 | -0.409 | 0.1594 | No |
| 475 | LIMA1 |  |  | 1899 | -0.421 | 0.1516 | No |
| 476 | E2F3 |  |  | 1907 | -0.429 | 0.1495 | No |
| 477 | KDELR2 |  |  | 1925 | -0.439 | 0.1418 | No |
| 478 | GGCT |  |  | 1930 | -0.442 | 0.1414 | No |
| 479 | GINS2 |  |  | 1932 | -0.445 | 0.1428 | No |
| 480 | CLIC1 |  |  | 1949 | -0.454 | 0.1357 | No |
| 481 | DENR |  |  | 1956 | -0.457 | 0.1343 | No |
| 482 | PHIP |  |  | 1992 | -0.483 | 0.1166 | No |
| 483 | FAM111A |  |  | 2002 | -0.497 | 0.1136 | No |
| 484 | TUBB6 |  |  | 2006 | -0.500 | 0.1141 | No |
| 485 | XPO1 |  |  | 2007 | -0.500 | 0.1163 | No |
| 486 | FABP5 |  |  | 2033 | -0.523 | 0.1044 | No |
| 487 | PPA1 |  |  | 2049 | -0.534 | 0.0982 | No |
| 488 | GGH |  |  | 2080 | -0.603 | 0.0838 | No |
| 489 | MIS18A |  |  | 2096 | -0.639 | 0.0781 | No |
| 490 | KIF5B |  |  | 2137 | -0.756 | 0.0587 | No |
| 491 | HIST1H1C |  |  | 2243 | -1.485 | 0.0057 | No |
Table: GSEA details [plain text format]

  

Fig 2: RICHARDS\_NOWAKOWSKI\_SCIENCE\_2017\_ORG\_UPREG: Random ES distribution      
 Gene set null distribution of ES for **RICHARDS\_NOWAKOWSKI\_SCIENCE\_2017\_ORG\_UPREG**
